# Socio-economic and behavioral characteristics associated with COVID-19 vaccine hesitancy under a declared state of emergency in Japan

**DOI:** 10.1016/j.bbih.2022.100448

**Published:** 2022-03-23

**Authors:** Naho Suzuki, Tetsuya Yamamoto, Chigusa Uchiumi, Nagisa Sugaya

**Affiliations:** a Graduate School of Sciences and Technology for Innovation, Tokushima University, Tokushima, Japan; b Graduate School of Technology, Industrial and Social Sciences, Tokushima University, Tokushima, Japan; c Unit of Public Health and Preventive Medicine, School of Medicine, Yokohama City University, Yokohama, Japan

**Keywords:** COVID-19, Vaccination, Pandemic, Vaccine hesitancy

## Abstract

Evidence regarding coronavirus disease 2019 vaccination indicates that some people hesitate to be vaccinated, and previous studies demonstrate the variables that influence hesitancy to vaccinate. However, they have not limited the target population to areas where infection is prominent. This study aimed to clarify the characteristics of people living in these areas who hesitate to be vaccinated and recommend effective approaches to encourage vaccination.

The survey was conducted online between February 24 and March 1, 2021, during which the 2nd state of emergency was declared in Japan. The analytic sample comprised 17,582 unvaccinated individuals (mean age ​= ​48.6 ​± ​13.8, range ​= ​18–90 years). The *t*-test results indicate that current or past treatment for physical illness exerted a strong influence on vaccine hesitancy (*d*s ​= ​0.30). Similarly, multiple regression analyses revealed that understanding the importance and necessity for preventive behaviors had the greatest influence on the intention to vaccinate (β ​= ​0.48). Regarding recommendations to promote willingness to be vaccinated, our findings indicated that clear explanation of the reasons for the necessity for these behaviors and collaboration between representatives of various communities would effectively encourage vaccination.

## 1 Introduction

Coronavirus disease 2019 (COVID-19) vaccination has been promoted in many countries, and socioeconomic and behavioral variables have been reported to influence public hesitation to vaccinate. Previous studies revealed that women (Edwards et al., 2021), young people (Schwarzinger et al., 2021), and those with low trust in the government (Murphy et al., 2021) tend to hesitate to be vaccinated.

The infection rate of COVID-19 varies widely among regions, and it is particularly important promote vaccination in areas with severe outbreak. Hence, it is necessary to examine the intention to be vaccinated and the characteristics that influence this among people living in such areas. However, few studies have identified the factors specific to the areas with severe outbreaks, how people living in these areas feel about getting vaccinated and the factors that influence them.

Therefore, focusing on people living in areas where the outbreak was severe and who were covered by the declared state of emergency, this study aimed to clarify the characteristics of people who hesitate to be vaccinated and to recommend effective approaches to encourage vaccination based on those characteristics.

## 2 Methods

### 2.1 Participants

In this survey, 17,911 participants (9,665 males and 8,246 females) were included for analyses. The survey was conducted online between February 24 and March 1, 2021, during which the 2nd state of emergency was declared in Japan. Participants were recruited from ten prefectures covered by the 2nd state of emergency in Japan. The exclusion criteria were as follows: age <18 years, high school students, and residents living outside the ten prefectures.

Data were collected on an online platform (Macromill, Inc., Tokyo, Japan). All participants voluntarily responded to the anonymous survey and provided informed consent. These data are the same as those collected by Yamamoto et al. (2021). A part of the data of Yamamoto et al. (2021) were used. This study was approved by the Research Ethics Committee of the Graduate School of Social and Industrial Science and Technology, Tokushima University (approval number: 212).

### 2.2 Measurements

#### 2.2.1 Socio-demographic variables

Sociodemographic (e.g., Dror et al., 2020; Malik et al., 2020) and socioeconomic (Bertoncello et al., 2020) variables affect intentions to be vaccinated. Therefore, participants were surveyed regarding age, sex (male or female), marital status (married or unmarried), presence of children (yes, no), and household income (<¥2 million (approximately $18,000), ¥2–4 million, ¥4–6 million, ¥6–8 million, ¥8–10 million, ¥10–12 million, ¥12–15 million, ¥15–20 million, >¥20 million, unknown). They were also asked whether they were a healthcare worker, whether they were currently or had ever been treated for a physical illness, and whether they were currently or had ever been treated for a psychological illness.

#### 2.2.2 Variables associated with the prevention of COVID-19 (prevention variables)

A previous study reported that decisions about whether to get vaccinated or not are influenced by altruism (Shim et al., 2012); thus, that the impact of altruism must be considered. Information was collected on whether the participants continued preventive behaviors (e.g., wearing a mask, washing their hands, refraining from going out) (*Continuous prevention*), whether they took preventive behaviors altruistically to prevent infection of family members and others (*Altruistic*), whether they took preventive behaviors to avoid social criticism and pressure (*Prevention to avoid social criticism and pressure*), and whether they understood the importance and necessity for preventive behaviors (*Importance of prevention*). Each item was assessed using a seven-point scale (1 ​= ​completely disagree, 4 ​= ​neither agree nor disagree, 7 ​= ​completely agree). All the scales used in this survey, including this scale, were developed in conjunction with the implementation of this survey.

#### 2.2.3 Trust in COVID-19 information sources (information variables)

The intentions to be vaccinated varies depending upon the source of information regarding COVID-19 (Murphy et al., 2021); so that variable was also included. Information was collected on whether participants trusted COVID-19 related information from the government (*Confident in COVID-19 information from the government*) and whether they trusted COVID-19 related information from social media (e.g., Twitter, Facebook) (*Confident in COVID-19 information on social media*). Each item was assessed using a seven-point scale (1 ​= ​completely disagree, 4 ​= ​neither agree nor disagree, 7 ​= ​completely agree).

#### 2.2.4 Intention for vaccination

Participants’ intention to be vaccinated (*Vaccination intent*) was assessed using a seven-point scale (1 ​= ​no intention, 4 ​= ​neither intend nor do not intend, 7 ​= ​fully intend).

### 2.3 Statistical analyses

We calculated the percentage of those who were already vaccinated, the overall mean intent to vaccinate, and the percentage of intention to/or not to vaccinate. Data for those who were already vaccinated were excluded from subsequent analyses. We conducted *t*-tests to examine whether sociodemographic variables differed between intentions to vaccinate. Tukey's test was conducted to examine whether the degree of intention to vaccinate varied according to household income. Multiple regression analyses were performed with vaccination intent as the objective variable, age, and six *prevention variables* (Variables associated with the prevention of COVID-19) and *information variables* (Trust in COVID-19 information sources) as exploratory variables. Additionally, we conducted power analyses for *t*-tests and multiple regression analyses. If the power <0.8, the possibility of committing a type II error increases (Cohen, 1992).

For all tests, significance was set at α ​= ​0.05, two-tailed. RStudio version 3.6.0 (R Core Team, 2020) was used to perform analyses.

## 3 Results

Among the 17,911 participants, 59 (0.33%) had already been vaccinated. Data for these individuals were excluded and 17,852 unvaccinated individuals (mean age ​= ​48.6 ​± ​13.8, age range ​= ​18–90 years) were retained for analyses.

The overall mean of the score for intention to vaccinate was 4.37 (*SD* ​= ​1.86): the number of participants who answered “completely disagree (1)” to “somewhat disagree (3)” was 4,501 (25.2%), “neither agree nor disagree (4)” was 5,067 (28.4%), and “somewhat agree (5)” to “completely agree (7)” was 8,284 (46.4%). Result of the power analyses indicated that sex (*power* ​= ​0.99), marital status, presence of children, being a health care worker, currently receiving treatment for severe physical diseases, and previous treatment for severe physical diseases (*power*s ​= ​1) displayed powers >0.8, while current treatment for severe psychological illness (*power* ​= ​0.06) and previous treatment for severe psychological illness (*power* ​= ​0.52) were <0.8.

Descriptive statistics and *t*-test results are presented in Table 1. Being female, unmarried, childless, a non-healthcare worker, not receiving treatment for physical illness currently or in the past, and not receiving treatment for a psychological illness in the past were significantly associated with lower intentions to vaccinate (*p* ​< ​0.05). The largest effect sizes (*d*) were observed for current or past treatment for physical illness (*d*s ​= ​0.30), having children (*d* ​= ​0.28), being a healthcare worker (*d* ​= ​0.28), and being married (*d* ​= ​0.26).

**Table 1:** Descriptive statistics and *t*-test results about intentions to vaccinate.

Vaccination intent scores for each variable	*n*	*M*	*SD*	*t*	*d* [95%Cl]	*p*
**Sex**						
male	9,618	4.44	1.89	5.11	0.08 [0.05–0.10]	<0.001
female	8,234	4.30	1.81			
**Marital status**						
married	11,414	4.54	1.82	15.92	0.26 [0.22–0.29]	<0.001
unmarried	6,438	4.07	1.88			
**Presence of children**						
yes	10,043	4.59	1.82	18.29	0.28 [0.25–0.31]	<0.001
no	7,809	4.08	1.86			
**Healthcare worker (self)**						
yes	1,081	4.85	1.88	8.79	0.28 [0.21–0.34]	<0.001
no	16,711	4.34	1.85			
**Current treatment of severe physical diseases**						
yes	794	4.90	1.84	8.31	0.30 [0.23–0.37]	<0.001
no	17,058	4.34	1.85			
**Previous treatment of severe physical diseases**						
yes	1,268	4.88	1.87	10.37	0.30 [0.24–0.35]	<0.001
no	16,584	4.33	1.85			
**Current treatment of severe psychological diseases**						
yes	1,032	4.36	1.97	0.01	−0.01 [-0.07 – 0.06]	p ​= ​0.991
no	16,820	4.37	1.85			
**Previous treatment of severe psychological diseases**						
yes	1,821	4.45	1.91	2.15	0.05 [0.00–0.10]	<0.05
no	16,031	4.36	1.85			
**Household income**^a^						
< ​2.0 million	1,162	3.39	1.92	–	–	–
2.0–3.9 million	3,498	4.31	1.91	–	–	–
4.0–5.9 million	4,049	4.38	1.83	–	–	–
6.0–7.9 million	2,914	4.45	1.80	–	–	–
8.0–9.9 million	1,884	4.43	1.84	–	–	–
10.0–11.9 million	1,012	4.61	1.83	–	–	–
12.0–14.9 million	612	4.74	1.82	–	–	–
16.0–19.9 million	304	4.66	1.90	–	–	–
> ​20.0 million	187	4.80	1.84	–	–	–
unknown	2,230	4.20	1.83	–	–	–

a Tukey's test was conducted between each category.

Participants with an annual household income lower than 2 million yen had significantly lower intention to vaccinate than those from other income categories, with the largest difference being between the group with an annual household income of more than 20 million yen (<2.0 million: *M* ​= ​3.99; >20.0 million: *M* ​= ​4.80; *p* ​< ​0.001).

The results of the multiple regression analyses are shown in Table 2. Lower age (β ​= ​0.12, *p* ​< ​0.001), distrust of COVID-19 related information from the government (β ​= ​0.19, *p* ​< ​0.001), distrust of COVID-19 related information on social media (β ​= ​0.04, *p* ​< ​0.001), and not understanding the importance and necessity for preventive behaviors (β ​= ​0.48, *p* ​< ​0.001) significantly predicted lower intention to vaccinate. Among the variables that displayed a significant effect, the variable with the largest standardized partial regression coefficient value was understanding the importance of prevention.

**Table 2:** Results of the multiple regression analysis: Predictors of preferences for vaccination.

Predictor variables	*M*	*SD*	b	β	*t*	*p*
**Socio-Demographic variables**						
Age	48.6	13.8	0.02	0.12	21.56	<0.001
**Prevention variables**						
Continuous prevention	5.52	1.71	0.00	0.00	0.16	p ​= ​0.877
Altruistic	5.42	1.71	0.00	0.00	0.11	p ​= ​0.912
Prevention to avoid social criticism and pressure	4.99	1.80	0.01	0.01	1.85	p ​= ​0.065
Importance of prevention	4.95	1.67	0.54	0.48	59.52	<0.001
**Information variables**						
Confident in COVID-19 information from the government	4.15	1.59	0.22	0.19	25.65	<0.001
Confident in COVID-19 information on social media	3.39	1.62	0.05	0.04	6.86	<0.001

## 4 Discussion

In this study, we clarified the socio-demographic, prevention, and information variables associated with the hesitation to be vaccinated in areas and periods of severe COVID-19 spread under a declared state of emergency. Analysis of intention to vaccinate revealed that more than half of the participants were either hesitant or uninterested in getting vaccinated. This study was conducted in late February to early March 2021, immediately after the date (February 17, 2021) when vaccination was initiated in Japan. Our findings indicate that in areas with ongoing spread of COVID-19, many people were hesitate to receive vaccination at the start of COVID-19 vaccination in Japan.

Participants who were female, unmarried, childless, non-healthcare workers, not receiving treatment for physical illness currently or in the past, and who had not received treatment for a psychological illness in the past were less likely to be willing to receive the vaccination. Among these factors, the effect size for variables related to current or past treatment for physical illness was the largest. Similarly, previous research indicates that being free of chronic diseases was significantly associated with hesitation or refusal to vaccinate (Schwarzinger et al., 2021). Our results are also consistent with previous evidence (Edwards et al., 2021) that vaccination rated are lower for women than men. However, it should be noted that the effect size for sex was small in this study. This may be related to the timing of the survey. Side effects have been identified as one reason women hesitate to be vaccinated (Yoda and Katsuyama, 2021; Kadoya et al., 2021). However, vaccination had just begun in Japan when this study was conducted. Therefore, the side effects were not widely recognized, which may explain the relatively small effect size for sex in this study.

Participants with an annual household income lower than 2 million yen had a significantly lower intention to vaccinate than those from other categories of household income. Several studies have also reported a relationship between annual income and hesitancy to vaccinate (Khubchandani et al., 2021; Kadoya et al., 2021), suggesting that economic disparity also affects vaccination intentions. However, household income is affected by other factors such as family forms, hence, future studies should take these factors into account and examine the influence of economic status on intention to vaccinate.

Multiple regression analyses revealed that lower age, distrust of COVID-19 related information from the government, distrust of COVID-19 related information on social media, and not understanding the importance and the necessity for preventive behaviors were variables that significantly predicted lower intentions to vaccinate. In particular, understanding of the importance of and the necessity for preventive behaviors had the greatest influence on the intention to vaccinate. Freeman et al. (2021) also suggested that individuals who are hesitant to vaccinate tend to be unaware of the public health benefits of vaccination. Therefore, even under the state of emergency where the spread of infection was severe, it became clear that the major factor that made people hesitate to be vaccinated was the lack of understanding of the significance of preventive behaviors.

There are some limitations to this study. As mentioned above, this study used some of the data from Yamamoto et al. (2021), and because scales used in Yamamoto et al. (2021) were limited to those focused on mental health, we were unable to evaluate the impact of all risk factors, such as perceived risk of COVID-19, fear of COVID-19, and comorbid conditions. Additionally, we used scales that have not been examine reliability and validity in this study. In future studies, we must use established scales and examine their reliability and validity.

Despite these limitations, given the results of this survey, we can provide suggestions for effective promotion of vaccination. First, the group we recommend for target includes individuals who have never been treated for physical illness, unmarried individuals, childless individuals, and those with relatively low household incomes. In addition to promoting vaccination to a wide range of people, as in the past, focusing on certain people, such as those mentioned above, would assist in increasing vaccination rates. Moreover, understanding the importance and necessity for preventive behaviors had the strongest effect on intention to vaccinate. Therefore, in addition to encouraging preventive behaviors, clearly explaining why these behaviors are necessary would effectively encourage vaccination as a preventive behavior. However, our results also indicate that trust in the information provided by the government is a variable that influences intention to vaccinate. Thus, that collaborative efforts between representatives of various communities with the government to disseminate vaccine information may be more effective than information provided by only the government. This is the first study to examine vaccine hesitancy among residents of areas under a declared state of emergency in Japan. The results indicate that risk and protective factors must be taken into account in our efforts to promote vaccination. Further, the suggestions mentioned above may be useful for policy makers.

## Declaration of competing interest

The authors declare no competing interests.

## References

[bib1] Bertoncello C., Ferro A., Fonzo M., Zanovello S., Napoletano G., Russo F., Baldo V., Cocchio S. (2020 Jun 5). Socioeconomic determinants in vaccine hesitancy and vaccine refusal in Italy. Vaccines.

[bib2] Cohen J. (1992 Jul). A power primer. Psychol. Bull..

[bib3] Dror A.A., Eisenbach N., Taiber S., Morozov N.G., Mizrachi M., Zigron A., Srouji S., Sela E. (2020 Aug). Vaccine hesitancy: the next challenge in the fight against COVID-19. Eur. J. Epidemiol..

[bib4] Edwards B., Biddle N., Gray M., Sollis K. (2021). COVID-19 vaccine hesitancy and resistance: correlates in a nationally representative longitudinal survey of the Australian population. PLoS One.

[bib5] Freeman D., Loe B.S., Yu L.M., Freeman J., Chadwick A., Vaccari C., Shanyinde M., Harris V., Waite F., Rosebrock L., Petit A., Vanderslott S., Lewandowsky S., Larkin M., Innocenti S., Pollard A.J., McShane H., Lambe S. (2021). Effects of different types of written vaccination information on COVID-19 vaccine hesitancy in the UK (OCEANS-III): a single-blind, parallel-group, randomised controlled trial. Lancet Public Health.

[bib6] Kadoya Y., Watanapongvanich S., Yuktadatta P., Putthinun P., Lartey S.T., Khan M.S.R. (2021). Willing or hesitant? A socioeconomic study on the potential acceptance of COVID-19 vaccine in Japan. Int. J. Environ. Res. Publ. Health.

[bib7] Khubchandani J., Sharmam S., Price J.H., Wiblishauser M.J., Sharma M., Webb F.J. (2021). COVID-19 vaccination hesitancy in the United States: a rapid national assessment. J. Community Health.

[bib8] Malik A.A., McFadden S.M., Elharake J., Omer S.B. (2020 Sep). Determinants of COVID-19 vaccine acceptance in the US. EClinicalMedicine.

[bib9] Murphy J., Vallières F., Bentall R.P., Shevlin M., McBridem O., Hartman T.K., McKay R., Bennett K., Mason L., Gibson-Miller J., Levita L., Martinez A.P., Stocks T.V.A., Karatzias T., Hyland P. (2021). Psychological characteristics associated with COVID-19 vaccine hesitancy and resistance in Ireland and the United Kingdom. Nat. Commun..

[bib10] R Core Team (2020). R: a language and environment for statistical computing. https://www.R-project.org/.

[bib11] Schwarzinger M., Watson V., Arwidson P., Alla F., Luchini S. (2021). COVID-19 vaccine hesitancy in a representative working-age population in France: a survey experiment based on vaccine characteristics. Lancet Public Health.

[bib12] Shim E., Chapman G.B., Townsend J.P., Galvani A.P. (2012 Sep 7). The influence of altruism on influenza vaccination decisions. J. R. Soc. Interface.

[bib13] Yoda T., Katsuyama H. (2021). Willingness to receive COVID-19 vaccination in Japan. Vaccines.

[bib14] Yamamoto T., Uchiumi C., Suzuki N., Sugaya N., Murillo-Rodriguez E., Machado S., Imperatori C., Budde H. (2021 Aug 13). The influence of repeated mild lockdown on mental and physical health during the COVID-19 pandemic: a large-scale longitudinal study in Japan. medRxiv.

